# Post-Sterilization Physicochemical Characterization and Biological Activity of Cellulose Nanocrystals Coated with PDDA

**DOI:** 10.3390/molecules29235600

**Published:** 2024-11-27

**Authors:** Ashley Donato, Siddharth Nadkarni, Lakshay Tiwari, Serafina Poran, Rajesh Sunasee, Karina Ckless

**Affiliations:** Department of Chemistry and Biochemistry, State University of New York at Plattsburgh, Plattsburgh, NY 12901, USA; adona009@plattsburgh.edu (A.D.); snadk001@plattsburgh.edu (S.N.); ypora001@plattsburgh.edu (S.P.)

**Keywords:** cellulose nanocrystals, zeta potential, DLS, non-covalent coating, protein corona, biocompatibility, cytotoxicity, immune response

## Abstract

The rapid expansion of medical nanotechnology has significantly broadened the potential applications of cellulose nanocrystals (CNCs). While CNCs were initially developed for drug delivery, they are now being investigated for a range of advanced biomedical applications. As these applications evolve, it becomes crucial to understand the physicochemical behavior of CNCs in biologically relevant media to optimize their design and ensure biocompatibility. Functionalized CNCs can adsorb biomolecules, forming a “protein corona” that can impact their physicochemical properties, including alterations in particle size, zeta potential, and overall functionality. In this study, CNCs were coated with low (8500 Da)- and high (400,000–500,000 Da)-molecular-weight cationic polymer (poly(diallyldimethylammonium chloride—(PDDA) via non-covalent grafting, and their physicochemical characteristics, as well as their biological effects, were assessed in physiologically relevant media after sterilization. Our findings show that autoclaving significantly alters the physicochemical properties of CNC-PDDA, particularly when coated with low-molecular-weight (LMW) polymer. Furthermore, we observed that CNC-PDDA of a high molecular weight (HMW) has a greater impact on cell viability and blood biocompatibility than its LMW counterpart. Moreover, cellular immune responses to both CNC-PDDA LMW and HMW vary in the presence or absence of serum, implying that protein adsorption influences cell-nanomaterial recognition and their biological activity. This study provides valuable insights for optimizing CNC-based nanomaterials for therapeutic applications.

## 1. Introduction

The expansion of medical nanotechnology has significantly broadened the scope of applications for cellulose nanocrystals (CNCs). Initially explored as drug delivery platforms [1], CNCs are now being developed for advanced bio-imaging techniques [2] and pH-responsive sensing systems, among other innovative applications [3]. This increasing integration of nanotechnology in biomedicine, especially for drug delivery systemic applications, demands a thorough understanding of the physicochemical behaviors of nanomaterials, especially in biologically relevant media. Nanomaterials, such as CNCs, can interact with a diverse range of biomolecules in biological fluids, including proteins [4]. These interactions, commonly referred to as the “protein corona”, can significantly alter the physicochemical characteristics of nanomaterials, such as size, surface charge, and functionality, effectively giving the nanoparticles a new biological identity [5,6]. Such changes have important implications for their biological responses, including their cytotoxicity and immunogenicity. CNCs are cellulose-based nanomaterials that are good candidates for biomedical applications because their precursor, cellulose, is abundant, sustainable and accessible as well as presenting low cytotoxicity. In addition, CNCs present a remarkable strength and abundance of hydroxyl groups on their surface, making them very suitable for tunable chemical modifications [7,8] that can be tailored to specific biomedical applications. For instance, coating the negative surface of CNCs with cationic polymer enables the newly generated cationic nanomaterial assembly to interact with negatively charged biomolecules, such as DNA and or phospholipids in the membranes, which is advantageous for gene delivery and applications in gene therapy [9,10].

One of the primary challenges in using nanomaterials for biomedical purposes is maintaining their physicochemical stability, especially after these nanomaterials undergo sterilization processes. Sterilization is a process that ensures the materials are free from contaminants. The stability of PDDA-coated nanomaterials is particularly critical to investigate, as this coating is primarily maintained through weak intermolecular forces, such as electrostatic interactions, rather than the stronger, more stable covalent conjugation. Moreover, these weak forces are highly susceptible to disruption by environmental changes, such as variations in pH and ionic strength, similar to the processes observed in protein folding and denaturation [11]. In previous work, it was shown that sterilization processes, such as autoclaving, can modify the protein profiles of CNC-based nanomaterials compared to their non-sterilized (NS) or filtered counterparts [4,12]. These protein–nanoparticle interactions are key determinants in nanoparticle recognition by the immune system, potentially influencing immunoreactivity and the induction of immune responses [13,14]. In addition, the protein corona modulates how nanoparticles interact with blood cells, affecting hemolysis, platelet activation, nanoparticle uptake and the viability of endothelial cells, all of which are key determinants of nanoparticle biocompatibility [15,16].

In this study, we synthesized cationic CNCs via the non-covalent grafting of CNCs with cationic polymers (diallyldimethylammonium chloride) of two distinct molecular weights, low (8500 Da) and high (400,000–500,000 Da). Despite both polymers presenting the same chemical (structural) composition, they might have different biological properties. For instance, in a study by Naumenko et al. [17], PDDA with a molecular weight below 100,000 Da exhibits significantly lower cytotoxicity compared to PDDA with molecular weights between 400,000 and 500,000 Da. Such findings highlight the importance of considering molecular weight when evaluating nanomaterials’ biocompatibility and toxicity. Furthermore, given the potential biomedical applications of CNC-PDDA, including drug delivery, it is critical to ensure that these nanomaterials are contaminant-free post-synthesis. Sterilization methods are commonly applied to achieve this sterility; however, these processes can affect the physicochemical stability of nanomaterials as well as their interaction with biological media [18]. Hence, we assessed the physicochemical stability of CNC-PDDA following two widely used sterilization methods: autoclaving, which involves heat and pressure, and filtration, which involves some degree of pressure [19]. We examined the impact of these sterilization methods on CNC-PDDA particle size, surface charge using dynamic light scattering (DLS) and zeta potential measurements as well as protein interactions by protein gel silver saining. We also investigated the biological activity of these two CNC-PDDA using distinct methods. Our findings provide critical insights into the influence of molecular weight and sterilization techniques on the stability and biological interactions of CNC-based nanomaterials, facilitating the optimization of their biomedical applications [4,20,21].

## 2. Results

### 2.1. Physicochemical Characterization in Biological Relevant Media

**Figure 1:**
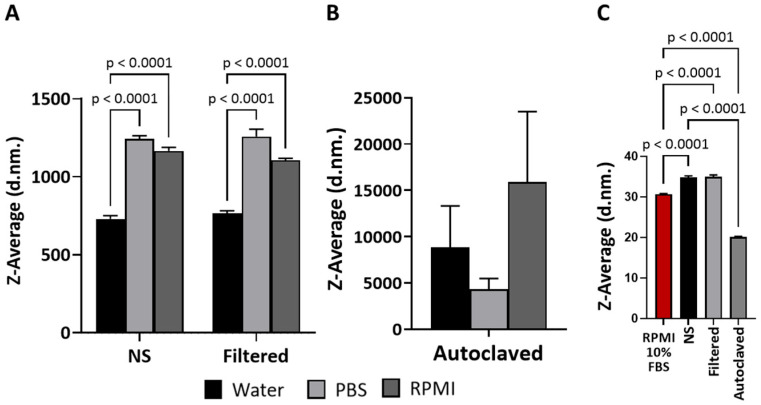
Apparent particle sizes of CNC-PDDA LMW suspensions. The non-sterile (NS) and filtered (**A**) and autoclaved (**B**) suspensions were prepared as described and exposed to relevant protein-free (**A**,**B**) and protein-rich (**C**) biological media, and apparent particle size (d.nm., diameter in nanometer) in the relevant biological media was assessed using a Malvern Zetasizer Nano-S instrument. Triplicates were measured for each sample, and averages and standard deviations are reported. ANOVA Tukey’s *p* < 0.05 compared to control or as indicated.

**Figure 2:**
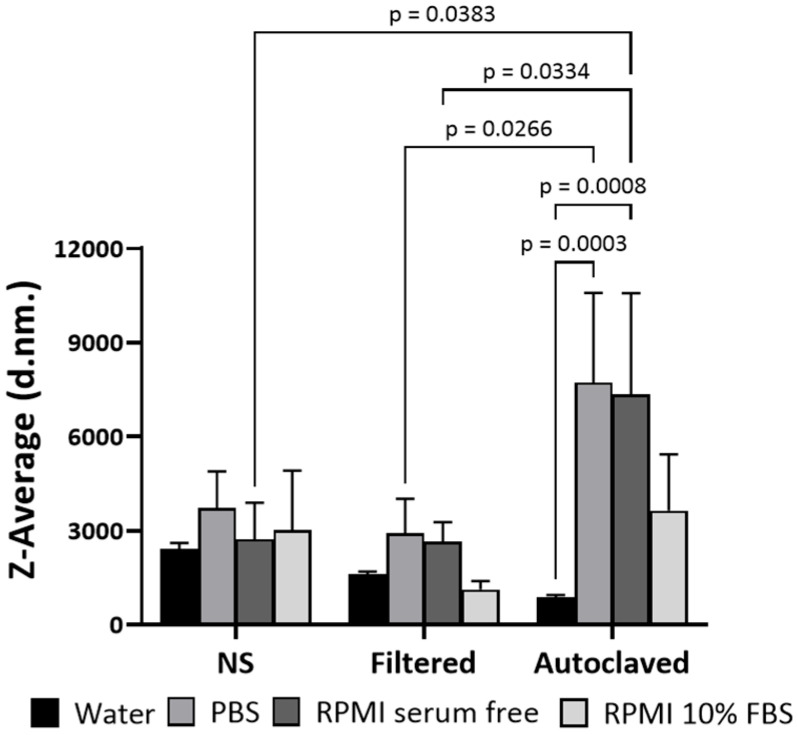
Apparent particle sizes of CNC-PDDA HMW suspensions. The non-sterile (NS), filtered and autoclaved suspensions were prepared as described and exposed to relevant protein-free (RPMI serum free) and protein-rich (RPMI 10% FBS) biological media, and apparent particle size (d.nm., diameter in nanometer) in the relevant biological media was assessed using a Malvern Zetasizer Nano-S instrument. Triplicates were measured for each sample, and averages and standard deviations are reported. ANOVA Tukey’s *p* < 0.05 compared to control or as indicated.

In this study, the nanomaterials were synthesized via the non-covalent grafting of PDDA of two distinct molecular weights onto the surface of CNC, and their colloidal solutions were prepared in ultrapure water. Subsequently, the solutions were subjected to two different sterilization methods, autoclaving and filtration, to evaluate their impact on their physicochemical characteristics in biological media. The apparent particle size, expressed as Z-average, of both CNC-PDDA was determined using dynamic light scattering (DLS). Both non-sterile (NS) and filtered CNC-PDDA exhibited similar behavior in water, PBS, and RPMI media with or without serum (Figure 1). Measurements in water (the reference medium) showed a lower Z-average compared to those in PBS and serum-free RPMI (Figure 1A). However, when CNC-PDDA LMW was autoclaved, the Z-average increased substantially compared to the NS and filtered samples, with no significant difference observed across the different media (Figure 1B). However, when the apparent particle size of CNC-PDDA LMW was assessed in RPMI medium containing 10% FBS, the overall apparent particle size of the preparations decreased substantially compared to the other media (Figure 1C). The non-sterile (NS) and filtered preparations of CNC-PDDA HMW showed no significant size differences between them and across all tested media. CNC-PDDA HMW exhibited a substantially higher Z-average in PBS and serum-free RPMI compared to the reference measurement in water. It was significantly higher than that of its filtered counterpart tested under the same conditions (Figure 2).

**Figure 3:**
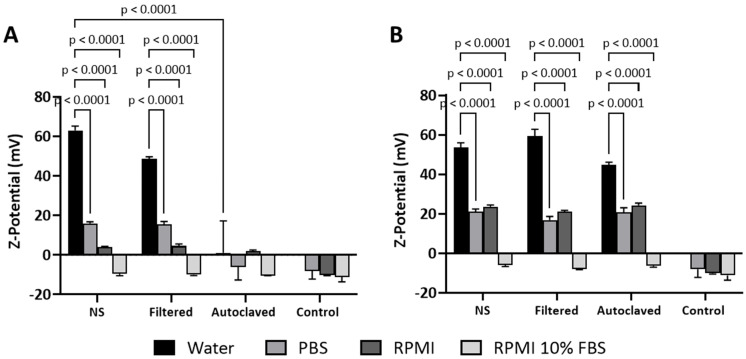
Zeta potential of (**A**) CNC-PDDA LMW and (**B**) CNC-PDDA HMW colloidal suspensions. The suspensions were prepared as described and exposed to relevant biological media, and Zeta potential (surface charges) was assessed using a Malvern Zetasizer Nano-S instrument. Triplicates were measured for each sample, and averages and standard deviations are reported. ANOVA Tukey’s *p* < 0.05 compared to control or as indicated.

We then evaluated the surface charges, expressed as zeta potential, of different preparations of both CNC-PDDA LMW and HMW in biological media. As expected, the NS and filtered preparations of CNC-PDDA LMW exhibited positive zeta potential when assessed in water. This zeta potential became progressively less positive when measured in PBS and serum-free RPMI, and the autoclaved preparation showed a significant decrease in the absolute value of zeta potential in all media. (Figure 3A). The behavior of CNC-PDDA HMW somehow differed from that of CNC-PDDA LMW. Surprisingly, autoclaving did not change the zeta potential of CNC-PDDA HMW. In fact, all preparations of CNC-PDDA HMW exhibited similar characteristics across the tested media (Figure 3B). Moreover, both CNC-PDDA LMW and HMW preparations showed lower positive zeta potential compared to water and negative zeta potential in RPMI 10% FBS, which is comparable to RPMI 10% FBS alone (Figure 3).

### 2.2. Protein-Nanomaterial Association

**Figure 4:**
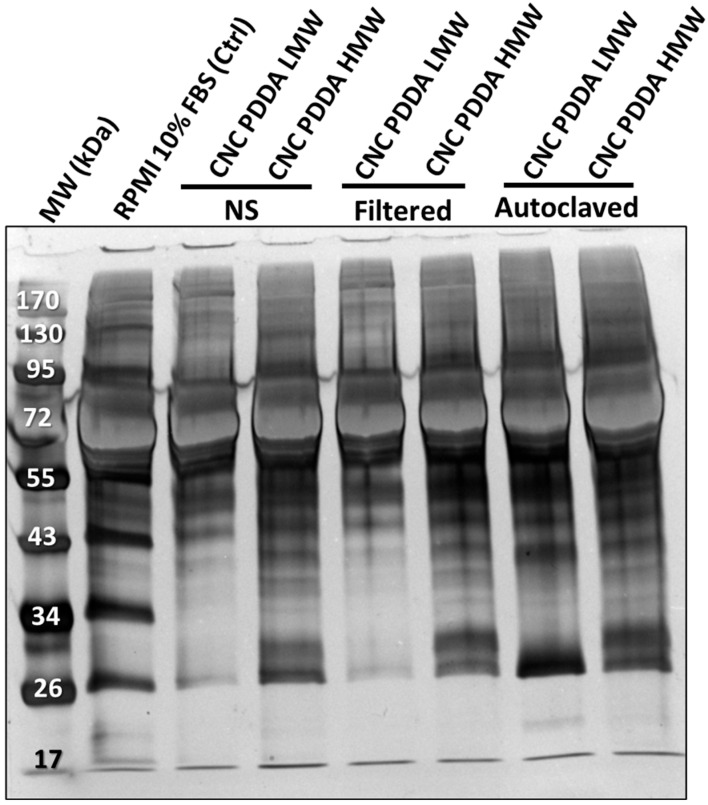
Association of serum protein with CNC-PDDA LMW and HMW. The CNC suspensions were prepared and mixed to RPMI 10% serum as described in methods. The samples were loaded onto a 10% sodium dodecyl sulphate polyacrylamide gel followed by electrophoresis (SDS-PAGE) at 150 V for 50 min. The gel was stained with SilverXpress^®^, according to the manufacturer’s instructions. The gel image was captured in the iBright gel imager. The numbers on the left are protein molecular weights (MW).

In line with these observations, both NS and filtered preparations of CNC-PDDA with low molecular weight (LMW) and high molecular weight (HMW) exhibited distinct protein interactions. However, these differences diminished significantly following the autoclaving process. Notably, HMW CNC-PDDA showed associations with a wider range of proteins compared to its LMW counterpart under the same conditions (Figure 4).

### 2.3. Cytotoxicity

**Figure 5:**
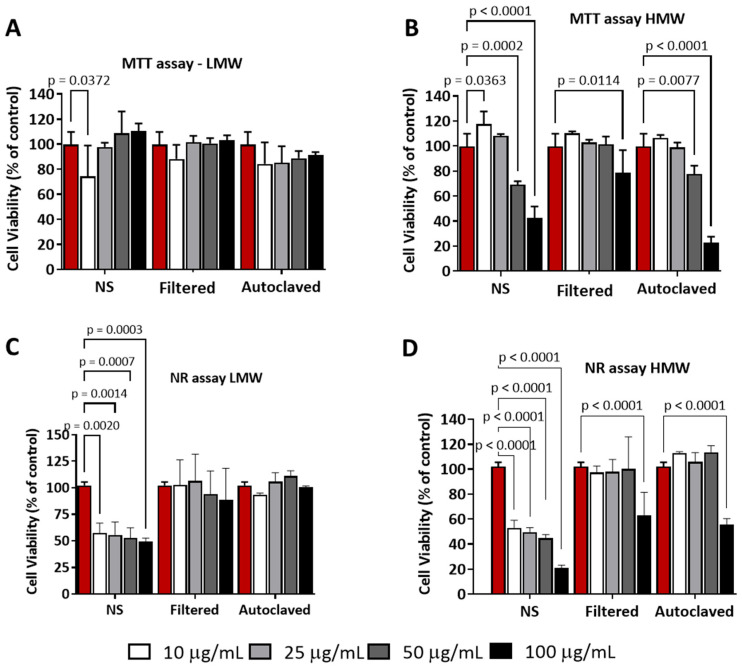
Effect of CNC-PDDA (LMW—(**A**,**C**) and HMW—(**B**,**D**)) on the cell viability of a mouse macrophage cell line (J774A.1). After 24 h of treatment in RPMI 10% FBS medium, cell viability was assessed using MTT (**A**,**B**) and NR (**C**,**D**) assay. Experiments were performed in triplicates. ANOVA Tukey’s *p* < 0.01 compared to control (100% cell viability), non-treated cells (red bars).

To explore the impact of these nanomaterials on cell availability, we assessed the cytotoxicity of various CNC-PDDA preparations using a mouse macrophage cell line (J774A.1, purchased from ATCC, Catalog number TIB-67) as the biological system, in the presence and absence of FBS (protein-rich medium) using two different colorimetric assays, MTT and neutral red (NR) viability assays. When cells were treated with RPMI 10% FBS, both CNC-PDDA LMW and HMW preparations affected cell viability, but in a very different manner, using MTT assay. None of the CNC-PDDA LMW formulations demonstrated a significant impact on cell viability (Figure 5A). In contrast, all CNC-PDDA HMW formulations markedly reduced cell viability at higher concentrations, with both NS and autoclaved HMW preparations showing a more pronounced cytotoxic effect (Figure 5B). In the Neutral Red (NR) assay, all CNC-PDDA LMW preparations led to a reduction in cell viability, particularly at the highest dose (Figure 5C). Similarly, all CNC-PDDA HMW preparations negatively affected cell viability, with the NS preparation exhibiting the most pronounced effect (Figure 5D).

**Figure 6:**
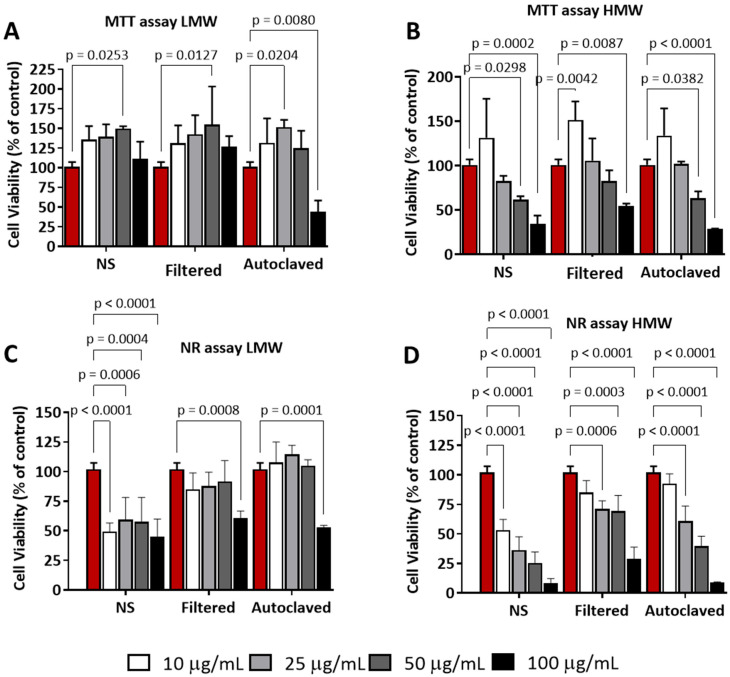
Effect of CNC-PDDA (LMW—(**A**,**C**) and HMW—(**B**,**D**)) on the cell viability of a mouse macrophage cell line (J774A.1). After 24 h of treatment in RPMI serum-free medium, cell viability was assessed using MTT (**A**,**B**) and NR (**C**,**D**) assays. Experiments were performed in triplicates. ANOVA Tukey’s *p* < 0.01 compared to control (100% cell viability), non-treated cells (red bars).

Furthermore, when cells in serum-free RPMI were treated with different preparations of both nanomaterials, a more pronounced reduction in cell viability was observed under most conditions. Once again, CNC-PDDA LMW had a lower impact on cell viability in both the MTT (Figure 6A) and NR assays (Figure 6C). In contrast, CNC-PDDA HMW demonstrated a clear dose-dependent effect on cell viability in both assays. Interestingly, in the MTT assay, the lowest concentration showed a slight tendency to increase cell viability, followed by a decrease (Figure 6B). In the NR assay, all CNC-PDDA HMW preparations consistently resulted in a gradual reduction in cell viability (Figure 6D). Collectively, these findings suggest that CNC-PDDA HMW exerts a more significant impact on cell viability, indicating higher toxicity compared to CNC-PDDA LMW, particularly in the absence of serum proteins (serum-free medium).

### 2.4. Blood Compatibility

**Figure 7:**
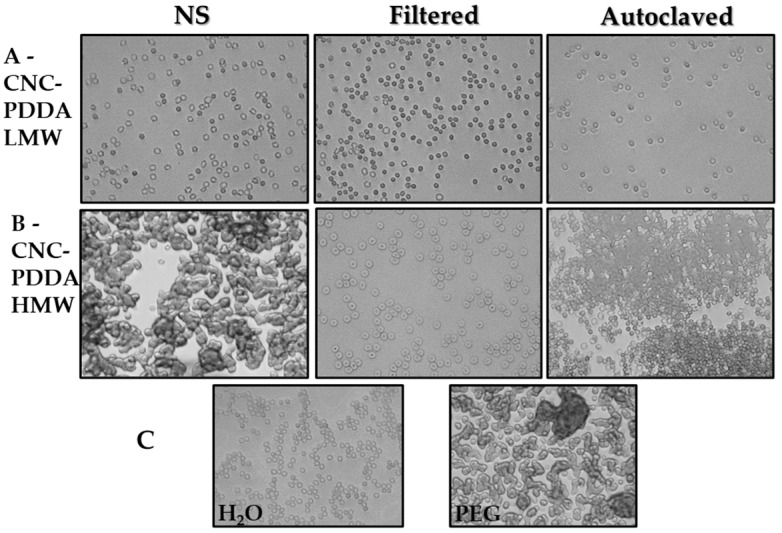
Effect of CNC-PDDA LMW (**A**) and HMW (**B**) on RBC morphology and aggregation. Diluted human blood was exposed for 24 h to 50 µg/mL of a colloidal suspension of nanomaterials as described in methods. Controls, ultrapure water (diluent) and PEG (1 mg/mL, positive control) are displayed in (**C**). The pictures were captured using a bright-field inverted microscope (400×).

**Figure 8:**
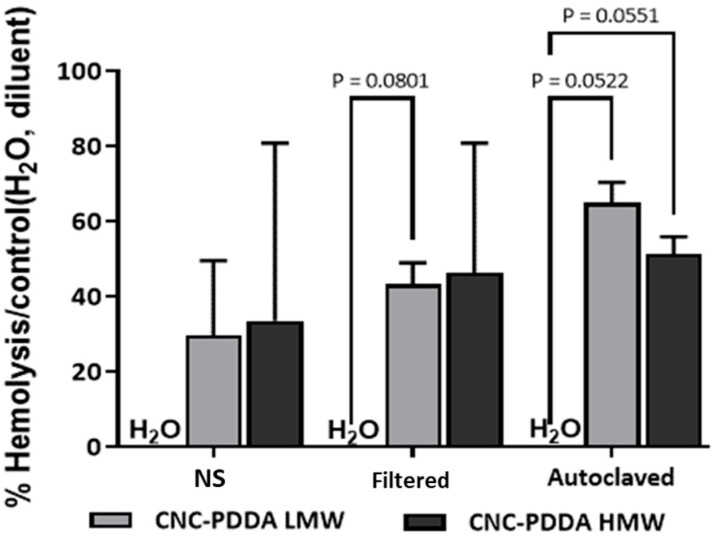
Hemolytic activity of CNC-PDDA LMW and HMW in human blood assessed using different sterilization methods. After 24 h of exposure of diluted whole human blood with 50 µg/mL of CNC-PDDA suspensions, the percentage of hemolysis was calculated by the Drabkin method using the hemolysis caused by ultra-pure water (diluent) as base-line hemolysis. PEG 1 mg/mL and Triton-X-100 1% were used as positive controls and caused 100% hemolysis. *p* < 0.1 vs. diluent ANOVA-Dunnett’s.

To assess this, we first examined whether CNC-PDDA LMW and HMW influence human red blood cell aggregation. We observed that none of the CNC-PDDA LMW preparations caused red blood cell (RBC) aggregation (Figure 7A) compared to the diluent, ultrapure water (Figure 7C, left panel). However, CNC-PDDA HMW induced significant RBC aggregation both in the non-sterilized preparations (Figure 7B, left panel) and after autoclaving (Figure 7B, right panel), similarly to the positive control, PEG (Figure 7C, right panel). Next, we evaluated the potential hemolytic effects of CNC-PDDA LMW and HMW preparations in human red blood cells. Hemolysis induced by the NS preparation of these nanomaterials was compared to the diluent, ultrapure water, which served as the baseline for hemolysis. However, significant hemolysis was observed with autoclaved preparations of both CNC-PDDA LMW and HMW, as well as with the filtered CNC-PDDA LMW preparation (Figure 8). While sterilized CNC-PDDA LMW preparations appeared to have a slightly greater impact on hemolysis compared to their HMW counterparts, they did not induce red blood cell (RBC) aggregation. In contrast, RBC aggregation was predominantly observed with CNC-PDDA HMW preparations. These findings are consistent with the observed effects on cell viability.

### 2.5. Immunogenicity

**Figure 9:**
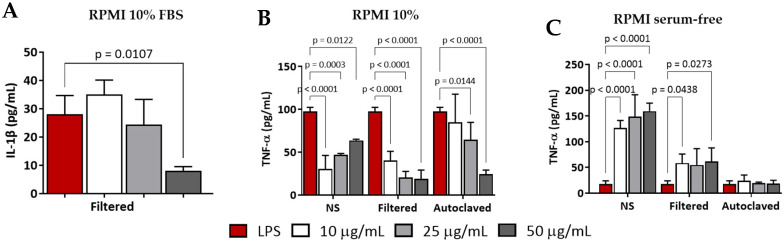
Immune response induced by CNC-PDDA LMW in LPS-stimulated (primed) mouse macrophages (J774A.1) in RPMI 10% FBS (**A**,**B**) and in serum-free medium (**C**). Primed-cells were treated with indicated concentrations of different preparations of CNC-PDDA LMW for 24 h. After treatments, the extracellular (supernatants) levels of IL-1β (**A**) and TNFα (**B**,**C**) were analyzed by ELISA. Statistical significance was determined by ANOVA—Tukey’s, *p* < 0.05 vs. LPS-treated cells. LPS = lipopolysaccharide.

In addition, we evaluated the potential immunogenicity of various preparations of both CNC-PDDA LMW and HMW using a primed mouse macrophage cell line (J774A.1). In a protein-rich medium (RPMI 10% FBS), filtered CNC-PDDA LMW significantly reduced IL-1β secretion at the highest dose (Figure 9A), whereas the other preparations did not affect IL-1β levels under these conditions. Additionally, different preparations of CNC-PDDA LMW reduced TNF-α secretion with the autoclaved preparation (Figure 9B). However, in the absence of serum, the effect on TNF-α secretion was reversed; both autoclaved and filtered CNC-PDDA LMW preparations led to increased TNF-α secretion (Figure 9C).

**Figure 10:**
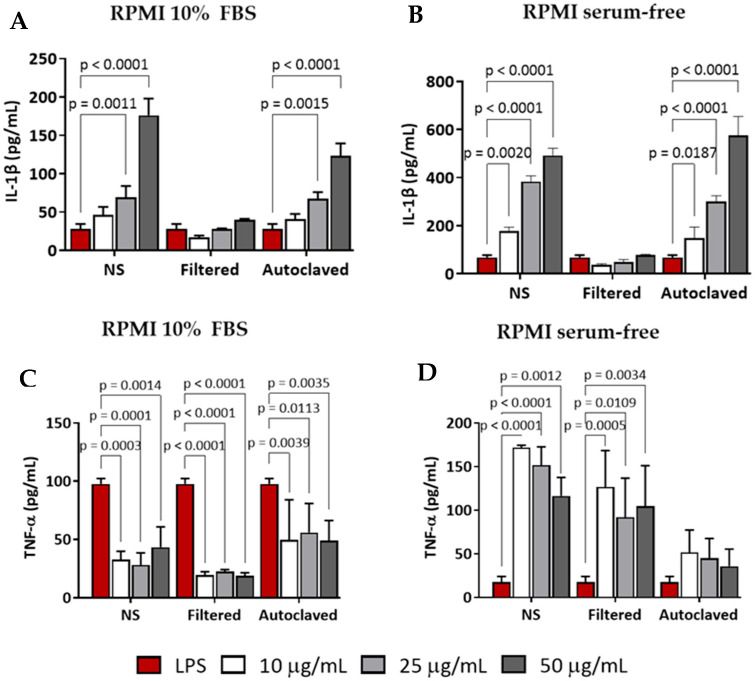
Immune response induced by CNC-PDDA HMW in LPS-stimulated (primed) mouse macrophages (J774A.1) in complete or incomplete cell culture medium. Primed-cells were treated with indicated concentrations of different preparations of CNC-PDDA LMW, for 24 h. After treatments, the extracellular (supernatants) levels of IL-1β (**A**,**B**) and TNFα (**C**,**D**) were analyzed by ELISA. Statistical significance was determined by ANOVA—Tukey’s, *p* < 0.05 vs. LPS-treated cells. LPS = lipopolysaccharide.

The immune response elicited by CNC-PDDA HMW in primed mouse macrophages was more consistent compared to its LMW counterpart. Both non-sterile and filtered CNC-PDDA HMW preparations increased IL-1β secretion in a dose-dependent manner, whether serum was present (Figure 10A) or absent (Figure 10B). Moreover, in the presence of serum, all CNC-PDDA HMW preparations also increased TNF-α secretion (Figure 10C). In contrast, in the absence of serum, all CNC-PDDA HMW preparations reduced TNF-α secretion; however, this effect was not dose-dependent (Figure 10D).

Overall, these data indicate that the presence of serum primarily influences the pattern of TNF-α secretion induced by both CNC-PDDA LMW and HMW. Specifically, serum appears to suppress TNF-α secretion, whereas in its absence, this suppression is reversed.

## 3. Discussion

Understanding the potential interactions between nanomaterials and biomolecules in biological fluids is critical for optimizing their biological activities and mitigating adverse effects. One critical aspect for the biomedical application of nanomaterials is ensuring their physicochemical stability, particularly when they undergo sterilization processes to achieve a contamination-free final product. Maintaining stability during and after sterilization is essential to preserve the functional properties and efficacy of nanomaterials in biomedical settings. In this study, we initially assessed the physicochemical properties of two CNC-PDDA nanomaterials in biologically relevant media.

Sterilization by autoclaving can affect the hydrodynamic size of nanomaterials, usually leading to an increase in size due to aggregation or particle interactions. Capping-based polymeric materials are also known to be more prone to size increases, aggregation and flocculation during autoclaving [22,23,24,25]. The aggregation phenomenon may partially explain the increase in size we observed in our PDDA-coated CNC materials following autoclaving. The observed decrease in size for CNC-PDDA LMW in RPMI medium supplemented with 10% FBS can be attributed to the ionic strength of the proteins in the medium. RPMI medium supplemented with 10% serum is considered protein-rich (=7 mg/mL proteins) [26]. These proteins may interact with and partially displace the LMW PDDA coating on the CNCs, leading to a reduction in particle size. This phenomenon was consistently observed across all preparations, including NS. However, it was not observed with the HMW counterpart, likely due to the higher-molecular-weight PDDA providing greater stability. The unusual zeta potential measurements of autoclaved CNC-PDDA LMW may indicate the decomposition of the low-molecular-weight (LMW) polymer coating on the CNCs due to thermal and pressure-induced changes during the autoclaving process. It has been reported that the activation energy for decomposition increases with the molecular weight of linear PDDA [21], suggesting that PDDA with a higher molecular weight (HMW) may offer greater stability during autoclaving compared to its LMW counterpart. Additionally, autoclaving has been demonstrated to reduce the absolute value of the zeta potential in silk fibroin nanoparticles [20]. This phenomenon has also been observed with other positively charged CNCs, where the cationic CNC preparations exhibited a negative zeta potential in RPMI medium with 10% FBS [4].

The significant alterations in the physicochemical characteristics observed in both CNC-PDDA LMW and CNC-PDDA HMW preparations under varying experimental conditions prompted us to further investigate potential differences in protein–nanomaterial interactions. Specifically, we focused on examining these associations in protein-rich cell culture medium (RPMI with 10% FBS). The protein–nanomaterial interactions, also called “corona”, occur when biomolecules, such as proteins in biological fluids, bind to the surface of nanoparticles (NPs), which can lead to alterations in the physicochemical characteristics of pristine nanomaterial [6,27,28]. In our previous work, we demonstrated that different nanomaterials interact with various proteins in cell culture medium containing fetal bovine serum (FBS). Specifically, we observed that autoclaving CNC-based nanomaterials altered their protein profiles compared to those of non-sterilized (NS) and filtered samples [4]. These observed differences in protein association related to polymer size correspond with previous studies indicating that smaller particle sizes and hydrophilic surfaces can effectively reduce protein adsorption [29]. It was also evident that the protein profile associated with CNC-PDDA HMW was less affected by different sterilization methods compared to CNC-PDDA LMW. Overall, the reduced impact on both the physicochemical properties and protein–nanomaterial interactions of CNC-PDDA HMW across different preparation methods reinforces the observation that this nanomaterial is more stable than its LMW counterpart, particularly when sterilized by autoclaving.

Proteins adsorbed onto nanoparticles can alter their intrinsic physicochemical properties, such as size, surface charge and functionality, effectively giving the nanoparticles a new biological identity. This newly formed identity plays a crucial role in shaping their biological responses, including cytotoxicity [28]. The PDDA molecular weight (MW)-dependent cytotoxicity observed in this study is consistent with findings from a recent investigation that evaluated the cytotoxicity of various cationic polyelectrolytes, including PDDA of different molecular weights, in human lung carcinoma cells (A549). The study demonstrated that PDDA with a molecular weight below 100,000 Da exhibited significantly lower cytotoxicity compared to PDDA with higher molecular weights (400,000–500,000 Da) [17].

Another critical aspect of nanomaterial compatibility for biomedical applications is their potential systemic effects, particularly their interactions with blood components and the downstream physiological consequences [15]. For potential biomedical application, it is also crucial to evaluate whether nanomaterials induce any undesired immune responses, since they interact with biomolecules in the biological fluids, and this can cause them to be more or less immunoreactive [13,14]. The observed differences in the immune responses to CNC-PDDA in serum-free versus RPMI 10% FBS conditions support the idea that the biological identity of CNC-PDDA is altered in the presence of serum, likely due to interactions between serum proteins and the CNC-PDDA nanoparticles. These interactions can influence immune cell recognition and response by modifying the surface properties of CNC-PDDA, independent of the molecular weight of the CNC-PDDA assemblies, leading to a decrease in TNF-α secretion. Conversely, in serum-free conditions, both CNC-PDDA nanoparticles remain uncovered and are more readily recognized by immune cells, resulting in increased TNF-α secretion. This effect, however, was not observed in IL-1β secretion, highlighting the pivotal role of serum proteins in modulating nanoparticle–immune cell interactions and influencing immune responses. Collectively, these findings emphasize the importance of serum proteins in shaping the immune-modulatory effects of CNC-PDDA nanomaterials.

## 4. Materials and Methods

### 4.1. Preparation and Characterization of CNC-PDDA

**Figure 11:**
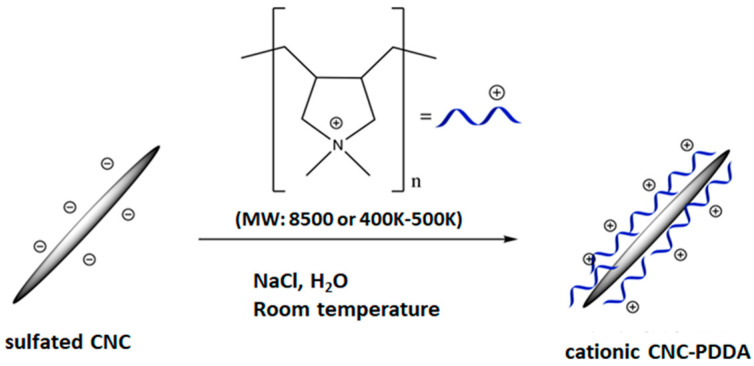
Schematic representation of the cellulose-based nanomaterial functionalized with two distinct molecular weights of poly(diallyldimethylammonium chloride (PDDA), 8500 (low molecular weight—LMW) and 400,000–500,000 (high molecular weight—HMW) respectively.

Spray-dried sulfated cellulose nanocrystals (CNC), derived from the acid hydrolysis of hardwood pulp, was non-covalently functionalized with 8500 Da (low molecular weight, LMW) or 400,000–500,000 Da (high molecular weight, HMW) of the cationic polymer, poly(diallyldimethyl ammonium chloride) (PDDA) using a modified reported method (Figure 11) [30,31]. PDDA (5 mL, 28 wt%, MW 8500 Da (LMW) or 400,000–500,000 Da (HMW)) was added to a suspension of CNC (2 mL, 5 wt%), and the mixture was sonicated for 30 min. The reaction mixture was then stirred for 24 h at room temperature, followed by the addition of NaCl (0.5 g). The mixture was further stirred for another 24 h at room temperature. The resulting suspension was diluted with deionized water, centrifuged and washed with deionized water several times. The suspension was freeze-dried to isolate CNC–PDDA LMW or CNC-PDDA HMW as a white solid. The CNC-PDDA nanomaterials were characterized by Fourier transform infrared spectroscopy (Figure S1, Supporting Information), dynamic light scattering, zeta potential (Table S1, Supporting Information) and elemental analysis (Table S2, Supporting Information).

### 4.2. Colloidal Suspensions Preparation of CNC-PDDA for Biological Assays

The colloidal suspensions of the CNC-PDDA LMW or HMW were prepared in ultra-pure water at 2 mg/mL, vortexed for 15 s, and sonicated for 2 min at 70% output. Post-sonication, the suspensions were filtered using a 0.45 µm polytetrafluoroethylene filter for debris removal. At this stage, the colloidal suspensions were deemed non-sterile. Next, one aliquot of non-sterile (NS) suspension underwent sterilization by autoclaving at 121 °C and 15 psi for 15 min and referred to as “autoclaved”, and another was submitted to additional filtration using a 0.22 µm nylon filter, named “filtered”. The resulting suspensions were aliquoted and stored at −20 °C for future biological assays.

### 4.3. Assessment of Physicochemical Characterization in Biological Relevant Media

A volume of 250 µL of nonsterile or sterile CNC-PDDA suspensions (2 mg/mL) were mixed with 750 µL of sterile phosphate-buffered saline (PBS, Gibco, Miami, FL, USA), sterile ultra-pure water (Millipore Milli-Q^®^ Direct 8 Water Purification System, Burlington, MA, USA ) or sterile RPMI (Gibco) with 10% fetal bovine serum (FBS, Gibco), penicillin-Streptomycin (10,000 U/mL Pen, 10 mg/mL Strep, Gibco) and L-glutamine (2 mM, Gibco), named RPMI 10% FBS or RPMI serum free. The medium-CNC-PDDA (LMW and HMW) mix, 1 mL total volume, was immediately vortexed and placed at 37 °C for 30 min followed by physicochemical analysis. The Zeta potential as well as dynamic light scattering (DLS) measurements of the CNCs’ colloidal suspensions were performed using a Malvern Zetasizer Nano ZS instrument (model: ZEN3600; Malvern Instruments Inc. (Malvern, UK)). The medium-CNC-PDDA (LMW and HMW) mix was vortexed for 10 s prior to the analysis. The DLS measurements were recorded six times, each in triplicate, and expressed as the average “apparent particle size” (d.m.). The polydispersity index (PDI) was also reported for each sample. The zeta potential measurements were performed in triplicate according to the manufacturer’s instructions, and the averages were documented.

### 4.4. Silver Staining and SDS-PAGE

The various colloidal suspensions of CNC-PDDA (LMW and HMW) mixed with RPMI 10% FBS were used to examine the nanomaterial–protein interactions. CNC-PDDA (LMW and HMW) and RPMI 10% FBS mix and RPMI 10% FBS medium alone (control) were centrifuged for 30 min at ~18,000× *g*, the supernatant with unbounded proteins was discarded, and the pellets re-suspended in PBS and sonicated to disrupt the pellet. Laemmli buffer was added to each sample followed by quick vortexing. Subsequently, the samples were heated at 90 °C for 5 min and loaded onto a 10% sodium dodecyl sulphate polyacrylamide gel followed by electrophoresis (SDS-PAGE) at 150 V for 50 min. The protein profile was detected using silver staining (SilverXpress^®^ Silver Staining, Invitrogen), according to the manufacturer’s directions. The protein profile on the gel was captured using a gel imager (iBright, BioRad Laboratories, Inc.: San Francisco, CA, USA).

### 4.5. Preparation of Human Blood

Human blood cells containing the anticoagulant citrate phosphate double dextrose solution (CP2D) were obtained from Leukotrap blood filters provided by healthy donors at UVM Health Network-CVPH North Country Regional Blood Center, Plattsburgh, NY. The blood cells were extracted by gradually flushing the filter once with a 50 mL air-filled syringe, yielding approximately 15 mL of blood. This blood was then diluted 1:10 in calcium and magnesium-free PBS to achieve a total hemoglobin concentration of approximately 1.5 mg/mL. The diluted blood (225 μL) was mixed with 25 μL of CNC-PDDA (LMW and HMW) suspensions, resulting in final concentrations of 25 and 50 μg/mL, in a 48-well plate. The mixture was incubated at 37 °C in a 5% CO_2_ environment for 24 h prior to analysis.

### 4.6. Evaluation of Red Blood Cell (RBC) Morphology and Aggregation

The morphological and aggregational changes in RBCs were observed using an Olympus CKX53 (Olympus America-Center Valley, PA, USA) inverted microscope equipped with a DP22 Olympus camera. Images were captured in bright field at 400× magnification.

### 4.7. Hemolysis Assay

The percentage of hemolysis was assessed by transferring 180 μL of the blood/CNC mixture to a sterile microtube and centrifuging it at approximately 1800× *g* for 10 min. After centrifugation, 1 mL of Drabkin reagent (Ricca, Arlington, TX, USA) was added to the supernatant and incubated for 10 min at room temperature in the dark. The absorbance was then measured spectrophotometrically at 540 nm. For each experimental condition, the total hemoglobin was determined by adding 1% Triton to 20 μL of the RBC/CNC mixture to fully release the hemoglobin. Drabkin reagent (1 mL) was then added to the wells, and the absorbance at 540 nm was recorded using a microplate spectrophotometer (Synergy H1 Hybrid Multi-Mode, BioTek/Agilent, Winoski, VT, USA). The controls included 1% Triton and 1 mg/mL PEG (positive), as well as ultra-pure water and PBS (negative). The blank was determined by measuring the absorbance of the Drabkin reagent at 540 nm, which was subtracted from the absorbance of all samples. The percentage of RBC lysis was then calculated using the following formula.

% lysis = [((Abs 540 nm of supernatants − Abs 540 nm of blank))/(Abs 540 nm of suspension − Abs 540 nm of blank) × dilution factor)] × 100

### 4.8. Cytotoxicity Assays

The effects of CNC-PDDA (LMW and HMW) on a mouse macrophage cell line (J774A.1) were assessed using the MTT assay (3-(4,5-dimethylthiazol-2-yl)-2,5-diphenyltetrazolium bromide, Sigma (Tokyo, Japan)) and the Neutral Red (NR) assay (Sigma). The MTT assay assesses the conversion of polar MTT (yellow) to non-polar MTT-formazan (blue/purple) by mitochondrial reductase [32]. The NR assay evaluates the ability of viable cells to uptake and bind the neutral red dye in lysosomes [33]. In both assays, cell viability is directly proportional to the concentration of the dye captured within the respective organelle. Briefly, J774A.1 cells were seeded in a 48-well plate at a density of 1–2 × 10^6^ cells/mL and cultured in complete medium overnight at 37 °C in a 5% CO_2_ environment. After overnight stabilization, the cells were treated 10–100 µg/mL of CNC-PDDA (LMW or HMW) in complete or plain RPMI. After treatment, the medium from the cell culture was discarded and replaced with 100 μL of fresh medium containing either 500 μg/mL of MTT or 50 μg/mL of NR. The cells were incubated at 37 °C in a 5% CO_2_ environment for 30 min. Following incubation, NR or MTT loading medium was removed, and the cells were washed once with PBS. Dimethyl sulfoxide (DMSO) was added to each well to solubilize the formazan crystals, and the absorbance was measured using a microplate spectrophotometer (Synergy H1 Hybrid Multi-Mode, BioTek/Agilent) at 570 nm. The NR dye was extracted from the lysosomes by adding 100 μL of desorb solution (1% glacial acetic acid, 50% ethanol) and shaking the plate for approximately 10–15 min in the dark. After shaking, the absorbance was measured at 540 nm and 690 nm using the microplate spectrophotometer. Non-treated cells (control) were considered as 100% cell viability in both assays. Both assays were performed in triplicate on three separate occasions for statistical significance.

### 4.9. PBMCs Preparation and Cell Culture Conditions for Immunological Assays

The human peripheral blood mononuclear cells (PBMCs) were withdrawn from Leukotrap blood filters provided by viable blood donors at UVM Health Network-CVPH North Country Regional Blood Center, Plattsburgh, NY, USA. The PBMC extraction involved carefully reverse-flushing the filter with 10 mL of calcium and magnesium-free PBS, repeated twice. The PBMC isolation was performed using a lymphocyte separation medium (LSM, MP Biologicals, Santa Ana, CA, USA). The PBMCs were utilized for immunological assays.

Mouse macrophage cell line (J774A.1, Sigma) and PBMCs were seeded in a 48-well plate at a density of 1–2 × 10^6^ cells/mL and cultured overnight at 37 °C in a 5% CO_2_ environment using complete RPMI medium. After overnight stabilization, the cells were either left non-primed or primed with 100 ng/mL LPS (Ultra-pure E. coli 0111 Lipopolysaccharide, InvivoGen (San Diego, CA, USA)) for 3–4 h. They were then treated with 10–50 μg/mL CNC-PDDA (LMW and HMW) for an additional 20 h. In experiments using plain RPMI, the complete medium was replaced with plain RPMI for all conditions before LPS priming. Cell treatments followed the same procedure as in the complete RPMI experiments.

The inflammatory cytokines interleukin-1 beta (IL-1β) and tumor necrosis factor-alpha (TNFα) secreted by J774A.1 cells were evaluated using enzyme-linked immunosorbent assay (ELISA) kits from BD Biosciences (San Jose, CA, USA) and R & D Systems (Minneapolis, MN, USA). The secretion of IL-1β and TNFα by PBMCs was also assessed using the ELISA kit from BD Biosciences. All ELISA assays were performed according to the manufacturer’s instructions.

### 4.10. Statistical Analysis

The results were statistically evaluated via two-way analysis of variance (ANOVA) and Tukey’s multiple comparison tests in the GraphPad Prism 9 software. Comparison groups and statistical significance (*p* < 0.05) are specified in the legend of the respective figures.

## 5. Conclusions

Surface modifications on nanomaterials can significantly impact their behavior in biological media. Hence, understanding the activity of nanomaterials in systemic circulation is crucial, as the protein corona modulates nanoparticle interactions with platelets and blood cells, impacting hemolysis, platelet activation, nanoparticle uptake and endothelial cell viability [16]. In the present study, we have investigated the physicochemical characterization in biological media and the biological activity of cellulose-based nanomaterials non-covalently functionalized with cationic PDDA of varying molecular weights. Our findings demonstrated that the physicochemical properties of CNC-PDDA, both low- and high-molecular-weight assemblies underwent significant alterations depending on the sterilization technique and the medium in which they were analyzed. Specifically, autoclaving might be an unsuitable sterilization method for PDDA-coated CNCs, particularly those coated with low-molecular-weight PDDA, due to noticeable changes in their stability. Moreover, we observed that the inflammatory response of cells to CNC-PDDA HMW appeared environment-dependent, differing notably in the presence or absence of serum. This indicated that protein adsorption on the nanomaterials might influence cell–nanomaterial interactions. Overall, these results emphasize the importance of understanding nanomaterial stability and interactions with biological systems—particularly protein binding, cytotoxicity, and immune responses—to ensure safe and effective biomedical applications.

## Data Availability

Data are contained within the article and Supplementary Materials.

## Supplementary Materials

The following supporting information can be downloaded at: https://www.mdpi.com/article/10.3390/molecules29235600/s1, Figure S1. FTIR analysis of sulfated CNC (blue color), CNC-PDDA-LMW (red color) and CNC-PDDA-HMW (green color); Table S1. DLS and zeta potential measurement of sulfated CNC and CNC-PDDA samples; Table S2. Elemental analysis of sulfated CNC and CNC-PDDA samples.

## References

[1] Jackson J.K., Letchford K., Wasserman B.Z., Ye L., Hamad W.Y., Burt H.M. (2011). The use of nanocrystalline cellulose for the binding and controlled release of drugs. Int. J. Nanomed..

[2] Dong S., Roman M. (2007). Fluorescently labeled cellulose nanocrystals for bioimaging applications. J. Am. Chem. Soc..

[3] Nielsen L.J., Eyley S., Thielemans W., Aylott J.W. (2010). Dual fluorescent labelling of cellulose nanocrystals for pH sensing. Chem. Commun..

[4] Nguyen H., Nguyen H.P.M., Bernier A., Chandradat R., Sunasee R., Ckless K. (2023). Impact of physiological media and sterilization methods on the physicochemical characteristics of engineered CNCs, and the effects on nanomaterial-protein interactions and immunological activity. Colloid. Interface Sci. Commun..

[5] Marichal L., Klein G., Armengaud J., Boulard Y., Chedin S., Labarre J., Pin S., Renault J.P., Aude J.C. (2020). Protein Corona Composition of Silica Nanoparticles in Complex Media: Nanoparticle Size does not Matter. Nanomaterials.

[6] Cedervall T., Lynch I., Lindman S., Berggård T., Thulin E., Nilsson H., Dawson K.A., Linse S. (2007). Understanding the nanoparticle-protein corona using methods to quantify exchange rates and affinities of proteins for nanoparticles. Proc. Natl. Acad. Sci. USA.

[7] Sunasee R., Barchi J.J. (2021). Nanocellulose: Preparation, Functionalization and Applications. Comprehensive Glycoscience.

[8] Sunasee R., Hemraz U.D., Ckless K. (2016). Cellulose nanocrystals: A versatile nanoplatform for emerging biomedical applications. Expert. Opin. Drug Deliv..

[9] Cai X., Dou R., Guo C., Tang J., Li X., Chen J., Zhang J. (2023). Cationic Polymers as Transfection Reagents for Nucleic Acid Delivery. Pharmaceutics.

[10] Abdelhamid H.N., Mathew A.P. (2022). Cellulose-Based Nanomaterials Advance Biomedicine: A Review. Int. J. Mol. Sci..

[11] Zhou H.X., Pang X. (2018). Electrostatic Interactions in Protein Structure, Folding, Binding, and Condensation. Chem. Rev..

[12] Bernier A., Tobias T., Nguyen H., Kumar S., Tuga B., Imtiaz Y., Smith C.W., Sunasee R., Ckless K. (2021). Vascular and Blood Compatibility of Engineered Cationic Cellulose Nanocrystals in Cell-Based Assays. Nanomaterials.

[13] Panico S., Capolla S., Bozzer S., Toffoli G., Dal Bo M., Macor P. (2022). Biological Features of Nanoparticles: Protein Corona Formation and Interaction with the Immune System. Pharmaceutics.

[14] Dobrovolskaia M.A., Shurin M., Shvedova A.A. (2016). Current understanding of interactions between nanoparticles and the immune system. Toxicol. Appl. Pharmacol..

[15] Jeong H., Hwang J., Lee H., Hammond P.T., Choi J., Hong J. (2017). In vitro blood cell viability profiling of polymers used in molecular assembly. Sci. Rep..

[16] Kyriakides T.R., Raj A., Tseng T.H., Xiao H., Nguyen R., Mohammed F.S., Halder S., Xu M., Wu M.J., Bao S. (2021). Biocompatibility of nanomaterials and their immunological properties. Biomed. Mater..

[17] Naumenko E., Akhatova F., Rozhina E., Fakhrullin R. (2021). Revisiting the Cytotoxicity of Cationic Polyelectrolytes as a Principal Component in Layer-by-Layer Assembly Fabrication. Pharmaceutics.

[18] Bernal-Chavez S.A., Del Prado-Audelo M.L., Caballero-Floran I.H., Giraldo-Gomez D.M., Figueroa-Gonzalez G., Reyes-Hernandez O.D., Gonzalez-Del Carmen M., Gonzalez-Torres M., Cortes H., Leyva-Gomez G. (2021). Insights into Terminal Sterilization Processes of Nanoparticles for Biomedical Applications. Molecules.

[19] Galicia-Gonzalez R.A., Ortega-Cerrilla M.E., Nava-Cuellar C., Miranda-Jiménez L., Ramírez-Mella M., Ayala-Rodríguez J.M. (2021). Nanoparticle sterilization methods for biomedical applications in animals. Agro Product..

[20] Asensio Ruiz M.A., Fuster M.G., Martinez Martinez T., Montalban M.G., Cenis J.L., Villora G., Lozano-Perez A.A. (2022). The Effect of Sterilization on the Characteristics of Silk Fibroin Nanoparticles. Polymers.

[21] Jia X., Zhan X., Xie J., Gao B., Zhang Y. (2019). Thermal stability of poly(diallyldimethylammonium chloride) with different molecular weight. J. Macromol. Sci. Part A.

[22] Bos G.W., Trullas-Jimeno A., Jiskoot W., Crommelin D.J., Hennink W.E. (2000). Sterilization of poly(dimethylamino) ethyl methacrylate-based gene transfer complexes. Int. J. Pharm..

[23] França A., Pelaz B., Moros M., Sánchez-Espinel C., Hernández A., Fernández-López C., Grazú V., de la Fuente J.M., Pastoriza-Santos I., Liz-Marzán L.M. (2010). Sterilization matters: Consequences of different sterilization techniques on gold nanoparticles. Small.

[24] Özcan I., Bouchemal K., Sánchez F., Abaci Ö.T., Özer Ö., Güneri T., Ponchel G. (2009). Effects of sterilization techniques on the PEGylated poly (γ-benzyl-L-glutamate) (PBLG) nanoparticles. Acta Pharm. Sci..

[25] Sommerfeld P., Schroeder U., Sabel B.A. (1998). Sterilization of unloaded polybutylcyanoacrylate nanoparticles. Int. J. Pharm..

[26] Hong X., Meng Y., Kalkanis S.N. (2016). Serum proteins are extracted along with monolayer cells in plasticware and interfere with protein analysis. J. Biol. Methods.

[27] Bertrand N., Grenier P., Mahmoudi M., Lima E.M., Appel E.A., Dormont F., Lim J.M., Karnik R., Langer R., Farokhzad O.C. (2017). Mechanistic understanding of in vivo protein corona formation on polymeric nanoparticles and impact on pharmacokinetics. Nat. Commun..

[28] Palchetti S., Digiacomo L., Pozzi D., Peruzzi G., Micarelli E., Mahmoudi M., Caracciolo G. (2016). Nanoparticles-cell association predicted by protein corona fingerprints. Nanoscale.

[29] Wang W., Huang Z., Li Y., Wang W., Shi J., Fu F., Huang Y., Pan X., Wu C. (2021). Impact of particle size and pH on protein corona formation of solid lipid nanoparticles: A proof-of-concept study. Acta Pharm. Sin. B.

[30] Dong L., Zhang X., Ren S., Lei T., Sun X., Qi Y., Wu Q. (2016). Poly(diallyldimethylammonium chloride)–cellulose nanocrystals supported Au nanoparticles for nonenzymatic glucose sensing. RSC Adv..

[31] Rabia E., Tuga B., Ondarza J., Ramos S.M., Lam E., Hrapovic S., Liu Y., Sunasee R. (2023). Carboxylated Cellulose Nanocrystals Decorated with Varying Molecular Weights of Poly(diallyldimethylammonium chloride) as Sustainable Antibacterial Agents. Polymers.

[32] van Meerloo J., Kaspers G.J., Cloos J. (2011). Cell sensitivity assays: The MTT assay. Methods Mol. Biol..

[33] Repetto G., del Peso A., Zurita J.L. (2008). Neutral red uptake assay for the estimation of cell viability/cytotoxicity. Nat. Protoc..

