# Supplementary material for: Post-Sterilization Physicochemical Characterization and Biological Activity of Cellulose Nanocrystals Coated with PDDA
Source: Molecules. 2024 Nov 27;29(23):5600. doi: 10.3390/molecules29235600 (PMC11643580; doi:10.3390/molecules29235600)
Supplement: Supplementary file 1 [file molecules-29-05600-s001.zip › molecules-3323147-supplementary.pdf]

molecules

Article

Post-Sterilization Physicochemical Characterization and Biological Activity of Cellulose Nanocrystals Coated with PDDA

Ashley Donato, Siddharth Nadkarni, Lakshay Tiwari, Serafina Poran, Rajesh Sunasee * and Karina Ckless *

Department of Chemistry and Biochemistry, State University of New York at Plattsburgh, Plattsburgh, NY 12901, USA; adona009@plattsburgh.edu (A.D.); snadk001@plattsburgh.edu (S.N.); ltiwa001@plattsburgh.edu (L.T.); ypora001@plattsburgh.edu (S.P.)

* Correspondence: rsuna001@plattsburgh.edu (R.S.); kckle001@plattsburgh.edu (K.C.)

Characterization of CNC-PDDA samples

1. FTIR analysis

FTIR spectra of spray dried sulfated CNC, free PDDA and freeze-dried CNC-PDDA samples were conducted on a PerkinElmer FTIR spectrophotometer (Spectrum Two) (Norwalk, CT, USA) at room temperature. Spectra in the range of 4000–400 cm^{-1} were obtained with a resolution of 4 cm^{-1} by cumulating 32 scans. Sulfated CNC displayed typical IR peaks characteristic of cellulosic functional groups at 3000–3600 cm^{-1}, 1645 cm^{-1} and 900–1150 cm^{-1}corresponding to the -OH, -OH bending of water and -C-O-C vibrations, respectively. The characteristic stretching bands remained almost the same as for CNC-PDDA samples indicating that the coating of PDDA onto CNC did not change the chemical structure of CNC.

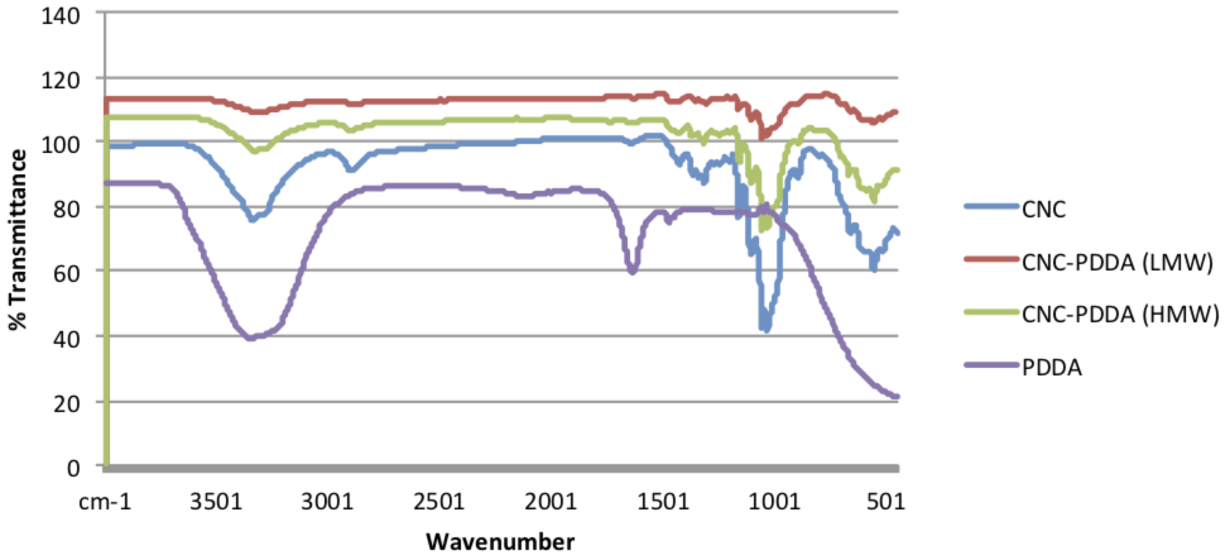

Figure S1. FTIR analysis of sulfated CNC (blue color), CNC-PDDA-LMW (red color), CNC-PDDA-HMW (green color) and PDDA (purple color).

2. Dynamic light scattering and zeta potential analysis

Dynamic light scattering (DLS) and zeta potential measurements of spray dried sulfated CNC and freeze-dried CNC-PDDA samples were obtained using a Malvern Zetasizer Nano ZS instrument (model: ZEN3600; Malvern Instruments Inc.,Westborough,

MA, USA). This instrument is equipped with a 4.0mW helium-neon laser and an avalanche photodiode detector and works at a 173° scattering angle. For DLS, the apparent particle size was measured for a 0.05 wt% CNC dispersion in Milli-Q water while a 0.25 wt% CNC dispersion in Milli-Q water was used for zeta potential measurements. Prior measurements, the suspensions were sonicated and filtered through a 0.45 µm filter membrane and analyses were performed immediately at 25 °C. For DLS results, triplicate samples were measured 12 times each and the average particle size distribution was obtained. The standard deviation was reported as the error for the measurements. Results for zeta potential measurements were recorded in triplicate and the averages were reported.

DLS results indicated an increase in apparent particle size for both CNC-PDDA samples, which confirmed the presence of the cationic PDDA on the surface of CNC. (Table S2). As expected, sulfated CNC had a negative zeta potential (-34.8 mV) due to the presence of anionic sulfate half-ester groups. However, after modification with cationic PDDA, CNC-PDDA samples displayed positive zeta potential values (+44.7 mV for CNC-PDDA-LMW and +53.6 mV for CNC-PDDA-HMW; Table S2). This further confirmed a successful non-covalent grafting of cationic PDDA on the surface of CNC that shielded the anionic sulfate half-ester groups.

Table S1. DLS and zeta potential measurement of sulfated CNC and CNC-PDDA samples.

Sample	Apparent Particle Size (nm)	Polydispersity Index (PdI)	Zeta Potential (mV)
Sulfated CNC	101.6 ± 0.7	0.23	-34.8 ± 2.2
CNC-LMW	163.1 ± 2.0	0.31	+44.7 ± 1.4
CNC-HMW	313.2 ± 5.4	0.27	+53.6 ± 2.4

3. Elemental analysis

The atomic composition (carbon, hydrogen, nitrogen, and sulfur content by mass) of CNC samples before and after PDDA grafting was measured using Thermo Scientific Flash 2000 Organic Elemental Analyzer. While sulfated CNC displayed the absence of nitrogen, both cationic CNC-PDDA samples indicated the appearance of nitrogen deriving from the cationic PDDA (Table S2). This further supported the success of the non-covalent grafting of PDDA onto CNC.

Table S2. Elemental analysis of sulfated CNC and CNC-PDDA samples.

Sample	% Carbon	% Hydrogen	% itrogen	% Sulfur
Pristine CNC	40.92	6.07	0.00	0.30
CNC-PDDA-LMW	43.28	6.54	1.20	0.00
CNC-PDDA-HMW	42.08	6.43	1.44	0.00
